# The first study on seroprevalence and risk factors for zoonotic transmission of ovine and caprine brucellosis in the Province of Bam, Burkina Faso

**DOI:** 10.14202/vetworld.2022.262-267

**Published:** 2022-02-05

**Authors:** Dieudonné Tialla

**Affiliations:** 1 Unit of Epidemic-Prone Diseases, Emerging Diseases and Zoonosis (UMEMEZ), National Influenza Reference Laboratory (LNR-G), Department of Biomedical and Public Health, Health Science Research Institute (IRSS), National Centre for Scientific and Technological Research (CNRST), Ouagadougou, Burkina Faso; 2 Department Animal Health, National School of Animal Husbandry and Health (ENESA), Ouagadougou, Burkina Faso

**Keywords:** brucellosis ovine and caprine, Burkina Faso, Province of Bam, seroprevalence, zoonosis

## Abstract

**Background and Aim:**

Brucellosis is a bacterial disease notorious for its ability to infect a wide range of domestic and wildlife animals, as well as humans. This study aimed to determine the seroprevalence of ovine and caprine brucellosis and the associated risk factors in the Province of Bam in Burkina Faso.

**Materials and Methods:**

The individual serological status of 300 unvaccinated sheep and 300 unvaccinated goats was determined by Rose Bengal and indirect enzyme-linked immunosorbent assay (iELISA) serological tests used in parallel. The frequency of behaviors conferring risk of developing this zoonotic disease was determined through two epidemiological questionnaires, which identified known risk factors for the transmission of brucellosis between animals and humans.

**Results:**

Individual seroprevalence was estimated at 6.0% (18/300) in sheep and 4.3% (13/300) in goats. The “herd” prevalence of brucellosis was estimated at 60% in sheep while 40% in goats. Positivity in the iELISA serological test was significantly associated with age, sex, and husbandry system in sheep and goats.

**Conclusion:**

These results indicate that *Brucella melitensis* circulates in sheep and goat farms in the Province of Bam in Burkina Faso. As *B. melitensis* is highly pathogenic to humans, adequate measures must be taken to protect the population against this zoonotic disease.

## Introduction

Brucellosis is a significant zoonosis most frequently caused by *Brucella melitensis*, *Brucella suis*, and *Brucella abortus*, which are highly pathogenic to humans [1-3]. It is very serious human disease in terms of public health and animal disease with extremely adverse economic and social consequences [4-6]. Indeed, it can lead to sterility and abortion, thus constituting a very serious problem for the health and well-being of animal populations [7-9]. It also hinders the marketing of animals and their products [5,10,11]. Females of dairy species excrete *B. melitensis* and *B. abortus* in their milk [12,13]. Moreover, *B. suis* remains a significant threat to populations exposed to domestic and wild pigs [14,15]. This genus may also be responsible for the formation of visceral abscesses in the elderly [16-18]. Despite recent progress in controlling this zoonotic disease, it remains common in urban, peri-urban, and rural areas of developing countries [19-21].

Ovine and caprine brucellosis is an infectious and highly contagious disease caused by *B. melitensis* [22,23]. It is the world’s first known zoonotic brucellosis [24-26]. It is highly pathogenic and may be the cause of late sheep abortions, ram orchitis-epididymitis, mastitis with milk loss, and complications (arthritis and nervous disorders) in sheep and goats [26,27]. It may also remain unapparent in the animal and even in the herd [28]. Compared with bovine brucellosis, ovine and caprine brucellosis is more easily transmitted to humans [22,23]. Indeed, ovine and caprine brucellosis is an animal disease that is contagious in all its forms [28,29]. Contamination usually occurs through unpasteurized milk and fresh cheese made with raw milk [22,23,30]. *Brucella* can also contaminate freshwater or vegetables grown in fields enriched with contaminated manure [18,31]. Inhalation contamination is most often found in enzootic zones among individuals in direct contact with animals, sheep wool, or manure. However, it may also arise in medical biology laboratories by handling an unidentified strain [28-30]. In fact, it is the most frequently acquired bacterial infection in laboratories [31]. Brucellosis can also be transmitted to humans through contact with the genital tract or with the aborted fetus after abortion of brucella origin [30,31].

Sheep and goat farming is a widespread agricultural practice in Burkina Faso and almost all rural households have at least one goat or sheep. However, to the best of our knowledge, nostudies on brucellosis in sheep and goats have been conducted in the country. Ovine and caprine brucellosis due to *B. melitensis* is highly pathogenic to humans. In this context, rural populations are exposed to the risk of contamination of this zoonosis. Hence, this study was established to assess the seroprevalence and behaviors conferring risk of zoonotic transmission of ovine and caprine brucellosis in the Province of Bam in Burkina Faso.

## Materials and Methods

### Ethical approval and informed consent

This study received the approval of the Institutional Ethical Committee from *Centre Muraz* (number: 2016-15/MS/SG/CM/IEC). Each farmer was informed and made aware of the objectives of the study. The written consent of each farmer was obtained before his involvement in the conduct of our study.

### Study period and location

The study was conducted from February 1, 2021, to July 20, 2021, in the Province of Bam. Located at the entrance of the Sahel at 110 km from the capital, the province is divided into eight departments including Kongoussi, Bouzanga, Guibaré, Nasseré, Rollo, Tikaré, Sabcé, and Zimtanga. Bam Province has a relief characterized by lateral plateaus and hill chains with a Sudanese-Sahelian climate and dry savanna vegetation.

### Study population and sampling method

The study population consisted of sheep and goats. Since the target population size was >10,000, our sample size was obtained using the following formula:

n=ε^2^×p×q/i^2^ [31],

Where n: Sample size; e=1.96: Narrow deviation for a 95% confidence interval (CI); p: Proportion of the target with the given character; q: 1–p; and i: Desired precision.

This is an estimate of the sample size with a maximum prevalence of 80% and a 95% confidence interval, which is a significance threshold of 5%. Recital p=0.8; q=0.2, and i=0.05, the minimum recommended sample size was 246. A two-stage random sampling method was used. The first stage involved the random selection of sheep and goat farms, while the second stage involved the random selection of sheep and goats from those farms. We conducted a preliminary survey since we did not have an exhaustive list of sheep and goat farms in our study area. This allowed us to identify 38 sheep and goat farmers meeting our inclusion criteria of having both an ovine and a caprine farm with at least 25 heads of each and agreeing to participate in this study. Of those 38 farmers, 20 were randomly selected. Thus, 40 farms, namely, 20 sheep farms and 20 goat farms, meeting the inclusion criteria, were randomly selected. The second stage involved the random selection of 15 animals per farm, or 300 sheep and 300 goats in total. Two visits to each farm were carried out: The first to inform the farmer about the study and obtain written consent of each and the second for blood sampling of the animals.

### Diagnostic methods

Blood samples (5 mL) were drawn from the jugular vein with dry tubes of 5 mL capacity. Each tube was identified by the farm code and the animal number. After centrifugation at 3600 rpm for 10 min [31], the sera were collected and placed in micro freezer tubes using sterile disposable pipettes. For the serological diagnosis of brucellosis, two serological tests were performed, namely, Rose Bengal and indirect enzyme-linked immunosorbent assay (iELISA), in accordance with the cold Kolmer technique [32]. The Bengal Rose test is a fast, simple, and cost-effective test that is considered sensitive (90%) and relatively non-specific (75%) [32]. The iELISA test is considered to be very sensitive (≥95%) and very specific (≥95%) [33]. The iELISA Kit (IDvet Innovative Diagnostics, France) enabled a search to be performed in 25 mL of serum for antibodies against *B. melitensis*, *B. abortus*, and *B. suis* by a microplate method, in accordance with the recommendations of the World Organization for Animal Health. The plates were read at 450 nm using a plate reader (Thermo Scientific Multiskan GO, Version 1.00.38). This made it possible to detect recent and old infections by highlighting immunoglobulin M and immunoglobulin G. The test was validated if the mean value of the positive control optical density (OD) (OD positive [ODP]) was >0.350 (ODP control [ODPC] >0.350), and the ratio of the mean OD values of the positive and negative controls (ODP and OD negative [ODN]) was >3.5 (ODP/ODN >3.5). In accordance with the manufacturer’s recommendations, positivity percentage (PP) was calculated using the following formula: PP=([OD sample/ODPC]×100). Samples with PP≤40% were considered negative, those between 40% and 50% were considered ambiguous, and those ≥50% were considered positive. The questionable cases were retested to determine their serological status better.

### Statistical analysis

The data were entered with EpiData^®^ software v4.2.0 (https://www.epidata.dk/) and processed with EpiData Analysis^®^ software v2.2.3 (https://www.epidata.dk/). The variable of interest, coded as presence/absence, was positivity in the iELISA laboratory diagnostic test. The explanatory variables were individual characteristics such as age, sex, and collective characteristics such as race, species. Comparisons of the different proportions were performed using the Chi-squared test and those of the averages were performed using the t-test and analysis of variance. Risk factors in sheep and goats and risk-conferring behaviors in humans were identified using a multivariate model. A logistic regression model (Proc Logistic, SAS^®^ System 9.3 v8.2) was used to analyze the positivity of the iELISA diagnostic test based on the explanatory variables considered as risk factors. The differences were considered statistically significant at p<0.05.

## Results

### Serological results of brucellosis in serum from 300 sheep collected in the Province of Bam in Burkina Faso

Table-1 presents the results of the brucellosis serological tests for sheep in this study. Out of 300 sera, 9 (3.0%) and 24 (8.0%) gave positive and ambiguous responses to Rose Bengal, respectively. After the analysis of these 33 samples using iELISA, the nine samples were positive with Rose Bengal and 24 samples with ambiguous Rose Bengal results gave positive responses. A total of 18 sheep (6.0%) showed a positive response to the iELISA test and 60% (12/20) of the farms showed at least one positive response to the Rose Bengal and iELISA tests.

**Table 1:** Test result for brucellosis on 300 sheep serums collected in the Province of Bam in Burkina Faso, 2021.

	iELISA positive	iELISA negative	Total
Rose Bengal positive	3% (9/300)	0% (0/300)	3% (9/300)
Rose Bengal doubtful	3% (9/300)	5% (15/300)	8% (24/300)
Rose Bengal negative	0% (0/300)	89% (267/300)	89% (267/300)
Total	6% (18/300)	94% (282/300)	100% (300/300)

iELISA=Indirect enzyme-linked immunosorbent assay

### Serological results of brucellosis in serum from 300 goats collected in the Province of Bam in Burkina Faso

Table-2 shows the results of the brucellosis serological tests for goats in this study. Out of 300 sera, 3 (1.0%) and 21 (7.0%) gave positive and ambiguous responses to Rose Bengal, respectively. After the analysis of these 24 samples using iELISA, the three samples positive with Rose Bengal and 21 samples with ambiguous Rose Bengal results gave positive responses. A total of 13 goats (4.3%) showed a positive response to the iELISA test and 40% (8/20) of the farms had at least one positive response to the Rose Bengal and iELISA tests.

**Table 2:** Test result for brucellosis on 300 serums of goats collected in the Province of Bam in Burkina Faso, 2021.

	iELISA positive	iELISA negative	Total
Rose Bengal positive	1% (3/300)	0% (0/300)	1% (3/300)
Rose Bengal doubtful	3.3% (10/300)	3.7% (11/300)	7% (21/300)
Rose Bengal negative	0% (0/300)	92% (276/300)	92% (276/300)
Total	4.3% (13/300)	95.7% (287/300)	100% (300/300)

iELISA=Indirect enzyme-linked immunosorbent assay

### Seroprevalence of brucellosis by age, sex, and breeding system of sheep collected from the Province of Bam in Burkina Faso, 2021

Positivity to the iELISA serologic test was significantly associated with age, sex, and rearing system. Table-3 presents the seroprevalence of brucellosis by age, sex, and breeding system of sheep collected from Bam Province in Burkina Faso, 2021.

**Table 3:** Seroprevalence of brucellosis by age, sex, and breeding system of sheep collected in the Province of Bam in Burkina Faso, 2021.

Variables	Sheep tested	Positive	Prevalence (%) and CI 95%	p-value
Age (years)				0.02
0-2	150	3	2.0±0.1	
>2	150	15	10.0±0.2	
Total	300	18	6.0±0.2	
Sex				0.01
Male	150	2	1.3±0.1	
Female	150	16	10.7±0.3	
Total	300	18	6.0±0.2	
Husbandry system				0.03
Intensive	150	13	8.7±0.2	
Extensive	150	5	3.3±0.1	
Total	300	18	6.0±0.2	

CI*=* Confidence interval

### Seroprevalence of brucellosis by age, sex, and breeding system of goats collected from the Province of Bam in Burkina Faso, 2021

Positivity in the iELISA serologic test was significantly associated with age, sex, and rearing system. Table-4 presents the seroprevalence of brucellosis by age, sex, and rearing system of goats collected in Bam Province in Burkina Faso, 2021.

**Table 4:** Seroprevalence of brucellosis by age, sex, and breeding system of goats collected in the Province of Bam in Burkina Faso, 2021.

Variables	Goats tested	Positive	Prevalence (%) and CI 95%	p-value
Age (years)				0.04
0-2	150	4	2.7±0.1	
>2	150	9	6.0±0.2	
Total	300	13	4.3±0.1	
Sex				0.02
Male	150	2	1.3±0.1	
Female	150	11	7.3±0.2	
Total	300	13	4.3±0.1	
Husbandry system				0.03
Intensive	150	10	6.7±0.2	
Extensive	150	3	2.0±0.1	
Total	300	13	4.3±0.1	

CI*=* Confidence interval

### Risk-conferring behaviors identified in humans

The most frequently observed risk-conferring behaviors among sheep and goat farmers in the Province of Bam in Burkina Faso were providing assistance with births and abortions, handling newborns without gloves, and consuming raw milk (Table-5).

**Table 5:** Risk behaviors observed among sheep and goat farmers in the Province of Bam in Burkina Faso, 2021.

Variables	OR	OR (CI: 95%)	p
Assistance in the delivery of calves	2.5	(2.3-2.7)	0.03
Assistance for abortions	2.8	(2.6-3.0)	0.02
Handling the runt without a glove	3.1	(2.9-3.3)	0.01
Consumption of unpasteurized raw milk	3.2	(3.0-3.4)	0.01

OR=Odds ratio; CI=Confidence interval

## Discussion

Vaccination against brucellosis is not carried out in Burkina Faso. In this country, individual seroprevalence was estimated at 6.0% in sheep and 4.3% in goats. These results are <7.7% in pigs and 18.3% in cattle previously reported in Burkina Faso [21]. They are also <15.87% reported among sheep in Egypt [34], while being similar to the individual seroprevalence of 4.1% obtained by Traoré *et al*. [35] in small ruminants in Mali. Meanwhile, our results are significantly higher than those obtained by Fediaevsky *et al*. [28], who found no antibodies against *B. melitensis* in serum collected from blood samples in sheep and goats in France. They pointed out that no infection with *B. melitensis* has been detected in France since the end of 2003. In addition, in 2012, Rautureau *et al*. [36] obtained the same results in France. However, they recommended that vigilance be maintained to maintain its status of being free from this zoonosis in sheep and goats. Our results are also higher than the rate of 0.48% obtained by Ebid *et a*l. [37]. Our results are also above 3.2% obtained in China [38]. These authors noted that the prevalence has been increasing over the years, as evidenced by an increase from 1% in 2000-2009 to 3.2% in 2010-2018. Contrary to our findings in the Province of Bam in Burkina Faso, their study showed that the prevalence of brucellosis was higher in goat herds than in sheep herds in China. Our results are also higher than the rate of 2% obtained by Crespo *et al*. [39] following an epidemiological study on brucellosis carried out on sheep and goats in Argentina. As in our study, they found that sheep had a higher rate of infection with *B. melitensis* than goats. In fact, they posited that sheep transmit the disease to goats. They, therefore, suggested avoiding the mixing of these two species to avoid recombination and genetic reassortment of the pathogen. Our results on the prevalence of brucellosis in sheep and goat are higher than those of Saeed *et al*. [29] and Edao *et al*. [40], with prevalence of 3.2% and 3.23%, respectively. These results show equal levels of infection and transmission of the pathogen to goats and sheep. This contrasts with our results showing that sheep are more affected than goats. Our results are also much lower than those reported in Pakistan for sheep and goats [41]. Indeed, these authors observed a prevalence of 75% in sheep and 61.5% in goats. This result was lower than the results obtained in Iraq [42] The latter estimated prevalence of 14.46% in sheep and 12.99% in goats. Climate, livestock hygiene, age, sex, and the applied rearing system could certainly influence the seroprevalence of brucellosis in sheep and goats. In fact, the positivity of the iELISA serological test was significantly associated with age, sex, and the breeding system in this study. Older sheep and goats were the most affected. Therefore, the risk of infection would increase with age. This seems logical because, the older the animal, the more likely it is to have been infected, remain infected, and be infectious to other animals. Moreover, females were found to be more infected than males. Generally, there are very few males in the herds and in addition, the females abort which is one of the remarkable and visible sign of brucellosis. Animals reared in intensive systems were the most affected. On the other hand, the conditions of extensive rearing limit the spread of brucellosis in contaminated herds. According to Traoré *et al*. [35], the rearing system can be considered a major risk factor in the transmission of brucellosis in sheep and goats. The contact between animals varies depending on the farming system. In the intensive system, the contacts are closer than in the extensive system.

The most common risk behaviors observed in humans were identified as assisting with births and abortions, handling newborns without gloves, and consuming raw unpasteurized milk. These behaviors pose a substantial risk regarding contact with the pathogen because ovine and caprine brucellosis is considered highly contagious, according to Fediaevsky *et al*. [28] and Saeed *et al*. [29]. Indeed, it is more easily transmitted to humans than bovine brucellosis [22,23]. This zoonotic transmission generally occurs through assisting with births and abortions, handling newborns without gloves, and consuming raw unpasteurized milk [30,35,37].

## Conclusion

This study determined the seroprevalence of ovine and caprine brucellosis and associated risk factors in the Province of Bam in Burkina Faso. It appears that, in this region, brucellosis is present in sheep and goat farms with seroprevalence of 6.0% and 4.3%, respectively. As *B. melitensis*, *B. suis*, and *B. abortus* are highly pathogenic to humans; adequate measures need to be taken to protect the population against this zoonosis. There is a need for raised awareness about biosecurity, the risks of zoonotic transmission of brucellosis, and the benefits of pasteurizing fresh and raw milk before its consumption among farmers and consumers.

## Authors’ Contribution

DT: Realization of all the steps of this study; conceptualization, preservation of data, formal analysis, surveys, laboratory analyses, statistical analyses, writing – original version, and, writing – revision and editing. The author read and approved the final manuscript.
